# Maternal Malaria and Malnutrition (M3) initiative, a pooled birth cohort of 13 pregnancy studies in Africa and the Western Pacific

**DOI:** 10.1136/bmjopen-2016-012697

**Published:** 2016-12-21

**Authors:** Holger W Unger, Jordan E Cates, Julie Gutman, Valerie Briand, Nadine Fievet, Innocent Valea, Halidou Tinto, Umberto d'Alessandro, Sarah H Landis, Seth Adu-Afarwuah, Kathryn G Dewey, Feiko Ter Kuile, Stephanie Dellicour, Peter Ouma, Laurence Slutsker, Dianne J Terlouw, Simon Kariuki, John Ayisi, Bernard Nahlen, Meghna Desai, Mwayi Madanitsa, Linda Kalilani-Phiri, Per Ashorn, Kenneth Maleta, Ivo Mueller, Danielle Stanisic, Christentze Schmiegelow, John Lusingu, Daniel Westreich, Anna Maria van Eijk, Steven Meshnick, Stephen Rogerson

**Affiliations:** 1 Department of Obstetrics and Gynaecology, Edinburgh Royal Infirmary, Edinburgh, UK; 2 Department of Medicine at the Doherty Institute, The University of Melbourne, Parkville, Victoria, Australia; 3 Department of Epidemiology, UNC-Chapel Hill, Chapel Hill, North Carolina, USA; 4 Malaria Branch, Division of Parasitic Diseases and Malaria, Center for Global Health, Centers for Disease Control and Prevention, Atlanta, Georgia, USA; 5 Institut de Recherche pour le Développement (IRD), Mère et enfant face aux infections tropicales (UMR216), Paris, France; 6 COMUE Sorbonne Paris Cité, Faculté de Pharmacie, Université Paris Descartes, Paris, France; 7 Unite de Recherche Clinique de Nanoro, Institut de Recherche en Sciences de la Santé-DRO, Bobo-Dioulasso, Burkina Faso; 8 Departement de Recherche Clinique, Centre Muraz, Bobo-Dioulasso, Burkina Faso; 9 Medical Research Council Unit, The Gambia; 10 London School of Hygiene and Tropical Medicine, UK; 11 Institute of Tropical Medicine, Antwerp, Belgium; 12 Worldwide Epidemiology, GlaxoSmithKline, Uxbridge, UK; 13 Department of Nutrition and Food Science, University of Ghana, Legon, Accra, Ghana; 14 Department of Nutrition, University of California, Davis, California, USA; 15 Department of Clinical Sciences, Liverpool School of Tropical Medicine, Liverpool, UK; 16 Kenya Medical Research Institute (KEMRI)/Center for Global Health Research, Kisumu, Kenya; 17 Malawi-Liverpool-Wellcome Trust Clinical Research Programme Liverpool School of Tropical Medicine, Liverpool, UK; 18 President's Malaria Initiative, Washington DC, USA; 19 School of Public Health and Family Medicine, College of Medicine, University of Malawi, Blantyre, Malawi; 20 Tampere Center for Child Health Research, Tampere, Finland; 21 Department for Pediatrics, University of Tampere and Tampere University Hospital, Tampere, Finland; 22 Walter and Eliza Hall Institute, Parkville, Victoria, Australia; 23 Institute for Glycomics, Griffith University, Gold Coast, Queensland, Australia; 24 Faculty of Health Science, Department of Immunology and Microbiology, Centre for Medical Parasitology, University of Copenhagen, Copenhagen, Denmark; 25 National Institute for Medical Research, Tanga Centre, Tanga, Tanzania

**Keywords:** malaria, malnutrition, pregnancy

## Abstract

**Purpose:**

The Maternal Malaria and Malnutrition (M3) initiative has pooled together 13 studies with the hope of improving understanding of malaria–nutrition interactions during pregnancy and to foster collaboration between nutritionists and malariologists.

**Participants:**

Data were pooled on 14 635 singleton, live birth pregnancies from women who had participated in 1 of 13 pregnancy studies. The 13 studies cover 8 countries in Africa and Papua New Guinea in the Western Pacific conducted from 1996 to 2015.

**Findings to date:**

Data are available at the time of antenatal enrolment of women into their respective parent study and at delivery. The data set comprises essential data such as malaria infection status, anthropometric assessments of maternal nutritional status, presence of anaemia and birth weight, as well as additional variables such gestational age at delivery for a subset of women. Participating studies are described in detail with regard to setting and primary outcome measures, and summarised data are available from each contributing cohort.

**Future plans:**

This pooled birth cohort is the largest pregnancy data set to date to permit a more definite evaluation of the impact of plausible interactions between poor nutritional status and malaria infection in pregnant women on fetal growth and gestational length. Given the current comparative lack of large pregnancy cohorts in malaria-endemic settings, compilation of suitable pregnancy cohorts is likely to provide adequate statistical power to assess malaria–nutrition interactions, and could point towards settings where such interactions are most relevant. The M3 cohort may thus help to identify pregnant women at high risk of adverse outcomes who may benefit from tailored intensive antenatal care including nutritional supplements and alternative or intensified malaria prevention regimens, and the settings in which these interventions would be most effective.

**Strengths and limitations of this study**

- This cohort pools data from 14 635 singleton, live birth pregnancies in women who participated in 1 of 13 pregnancy studies in areas with high burdens of malaria and undernutrition.
- The Maternal Malaria and Malnutrition (M3) pooled data set uses data collected at first antenatal attendance and at time of delivery.
- The data set comprises essential information on malaria infection status, maternal anthropometric indicators of undernutrition, presence of maternal anaemia, neonatal anthropometrics and gestational age at delivery, among others.
- The main limitation is the heterogeneity in availability of certain variables due to study design and data collection differences between studies.

## Introduction

It is estimated that each year over 125 million pregnant women residing in low-income and middle-income countries (LMICs) are at risk of infection with *Plasmodium falciparum* and *P. vivax*.^1^ Malaria contributes to the high burden of maternal morbidity and mortality in these settings and may affect placental development and fetal growth.^2^ Sequestration of *P. falciparum* parasites in the placenta has been associated with fetal growth restriction (FGR) and low birth weight (LBW, <2500 g), thus contributing to infant mortality and possibly long-term health problems.^3^ ^4^ *P. vivax* has also been associated with adverse pregnancy outcomes.^5^ Malaria during pregnancy may cause maternal anaemia, which itself can have deleterious effects on fetal development.^2^ Maternal undernutrition is also common among pregnant women in these settings,^6–8^ and undernourished women are more likely to have growth-restricted fetuses and babies with reduced birth weight.^9^ To date, it remains unclear whether the susceptibility to, and the impact of, malarial infection are affected by maternal nutritional status. A small number of studies suggest that macronutrient nutritional status modifies the effects of malaria in pregnancy, specifically the impact of *P. falciparum* parasitaemia on fetal growth and birth weight.^10–12^ Although largely overlooked, this may not be surprising given that both macronutrient and micronutrient nutritional status affect immune function more broadly.^13^ ^14^ As such, there may be scope to design interventions that prevent adverse pregnancy outcomes by protecting women and their offspring from the deleterious consequences of malaria and undernutrition. This aligns with the 2015 Global Strategy for Women's, Children's and Adolescents' Health recommendations to reduce the risk of LBW and maternal anaemia through the prevention and treatment of malaria and through adequate nutrition during pregnancy.^15^ ^16^

The Malaria in Pregnancy Consortium (MiPc), which receives funding from the Bill & Melinda Gates Foundation, brings together scientists whose aim is to reduce the burden and impact of malaria in pregnancy in LMICs.^17^ ^18^ At the 2014 MiPc meeting in New Orleans, Louisiana, USA, Consortium members identified the need to study the relationship between macronutrient nutritional status and malaria in pregnancy, given the high prevalence of undernutrition in malaria-endemic countries. The MiPc and other malaria researchers have conducted a number of malaria in pregnancy studies (observational studies and randomised controlled trials (RCTs) of malaria prevention strategies) and many had collected maternal anthropometrics and data on anaemia. Similarly, some recent trials of nutritional supplementation during pregnancy collected malariometric indices. Drawing on these data, there is a unique opportunity to study the interaction between undernutrition and malaria, together with other risk factors such as maternal anaemia.

The Maternal Malaria and Malnutrition (M3) initiative has pooled data from 13 studies with the hope of improving the understanding of malaria–nutrition interactions and to foster collaboration between nutritionists and malariologists. Reduction of LBW and anaemia were two of the six global nutrition targets for 2025 agreed on at the 2012 World Health Assembly.^15^ As of 2014, the Global Nutrition Report found that there was little progress towards this target.^19^ Risk factors for, and interventions to prevent, LBW are all too commonly studied in isolation and without cross-discipline collaboration. Topics of interest for M3 include an evaluation of whether macronutrient undernutrition modifies the impact of malaria infection during pregnancy; the investigation of potential interventions that address both malaria risk and nutritional status during pregnancy; and the study of the role of anaemia in relation to adverse pregnancy outcomes.

## Pooled cohort description

### Study populations

The M3 initiative comprises 13 studies (7 RCTs and 6 cohort studies) conducted among pregnant women in malaria-endemic countries from 1996 to 2015.^10^ ^20–32^ Studies were selected based on the availability of a predetermined set of essential variables for each woman, adequate ethical clearance and willingness of collaborators to share data. Essential variables included the assessment of malariometric indices (light microscopy (LM), quantitative PCR (qPCR) and/or rapid diagnostic tests (RDTs)) at enrolment/first antenatal care visit (ANC), assessment of anthropometric indicators at enrolment (mid-upper arm circumference (MUAC) and/or body mass index (BMI)), gravidity, type of malaria prevention used and maternal age. Essential delivery outcomes included birth weight and newborn sex. With birth weight being the primary focus, we restricted inclusion to women who delivered live singleton infants without congenital abnormalities, as previously recommended.^33^

**Figure 1:**
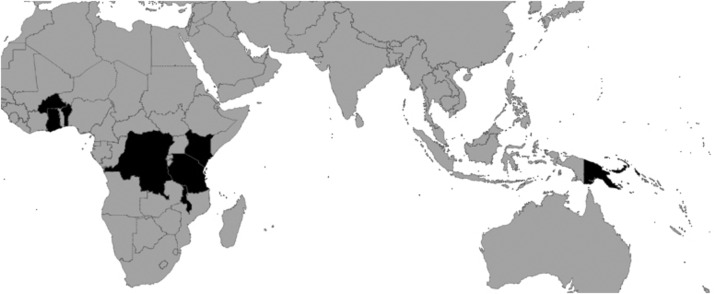
Geographical locations where the 13 parent studies were conducted: Benin, Burkina Faso, Democratic Republic of the Congo, Ghana, Kenya, Malawi, Papua New Guinea and Tanzania.

We included 14 635 singleton, live birth pregnancies from women who had participated in 1 of 13 pregnancy studies (table 1) conducted in seven countries in Africa and one in the Western Pacific (figure 1, table 1). Of the seven RCTs, four assessed the effectiveness of different pharmacological malaria prevention regimens,^27–30^ one additionally evaluated nutritional supplementation,^21^ one was designed solely to assess the effectiveness of nutritional supplementation^22^ and one measured the impact of insecticide-treated bed nets (ITNs) on birth weight.^25^ The six prospective cohort studies were all designed to assess risk factors and consequences of malaria infection and/or antimalarial treatment during pregnancy in different locations and among different study populations.^10^ ^20^ ^23^ ^26^ ^31^ ^32^

**Table 1:** Characteristics of the 13 individual studies included in the M3 initiative

Countries	Study name	Design	Period	Median GA (IQR)*	Malaria prevention†	Nutritional intervention	*N*‡	*n*§
Benin	STOPPAM I^20^	Cohort	2008–2010	17 (14–20)	IPT-SP	None	1037	791
BF	FSP/MISAME^21^ ^34^	RCT	2006–2008	16 (11–21)	IPT-SP(2), IPT-SP(3)	MMS, FFS	1296	1020
DRC	ECHO^10^	Cohort	2005–2006	19 (17–21)	IPT-SP	None	182	164
Ghana	iLiNS-DYAD^22^	RCT	2009–2012	17 (15–20)	IPT-SP	LNS, MMN, IFA	1320	1068
Kenya	EMEP and IPTp-MON^23^ ^24^	Cohort ¶	2011–2013	23 (16–30)	IPTp-SP	None	1453	473¶
Kenya	ITN^25^	RCT	1996–1999	24 (20–30)	IPT-SP started during study	None	911	711
Kenya	Kisumu cohort^26^	Cohort	1996–2001	36 (34–37)	IPT-SP started during study	None	3155	3388**
Kenya	STOPMIP^27^	RCT	2012–2015	23 (20–26)	IPT-SP, IPT-DHA-PQ, IST-DHA-PQ	None	1546	1203
Malawi	ISTp^28^	RCT	2011–2013	21 (19–23)	IPT-SP, IST-DP	None	1873	1602
Malawi	LAIS^29^	RCT	2003–2006	20 (18–23)	IPT-SP(2), IPT-SP(4) IPT-SPAZ	None	1320	1190
PNG	IPTp study^30^	RCT	2009–2013	22 (19–25)	IPT-SPAZ; Single dose SP and CQ	None	2793	1943
PNG	Sek cohort^31^	Cohort	2005–2007	25 (22–28)	Single dose SP and weekly CQ	None	470	293
Tanzania	STOPPAM II^32^	Cohort	2008–2010	19 (15–21)	IPT-SP	None	995	789

*Median (IQR): gestational age at enrolment assessed by fetal biometry, or symphysis-pubis fundal height when ultrasound unavailable.

†If RCT, describes the intervention, if cohort, describes the national policy during the study period.

‡N, enrolled in parent study.

§*n,* live birth pregnancies that met inclusion criteria for M3.

¶The EMEP study was a prospective cohort study with some overlapping enrolment with the cross-sectional study IPTp-MON. One hundred and eleven pregnancies were enrolled in EMEP and IPTp-MON; information on malaria infection at delivery was obtained for the subset of women in IPTp-MON.

**Includes additional women from a substudy not included in the parent study which otherwise met inclusion criteria for the pooled data.

BF, Burkina Faso; CQ, chloroquine; DHA-PQ, dihydroartemisinin-piperaquine; DRC, Democratic Republic of the Congo; FFS, fortified food supplementation; IFA, iron and folic acid supplementation; IPTp, intermittent preventive treatment in pregnancy; IST, intermittent screening for malaria infection; ISTp, intermittent screening for malaria infection in pregnancy; ITN, insecticide-treated bed net; LNS, lipid-based nutrient supplementation; M3, Maternal Malnutrition and Malaria; MMS, multiple micronutrients supplementation; PNG, Papua New Guinea; RCT, randomised controlled trial; SP, sulphadoxine-pyrimethamine; SPAZ, SP and azithromycin; STOPPAM, Strategies To Prevent Pregnancy Associated Malaria.

For 10 of the 13 studies, pregnant women were identified and recruited at one of their self-initiated ANC, usually the booking visit^10^ ^20^ ^22^ ^27–32^ but sometimes at a subsequent visit.^26^ The EMEP study enrolled women early in pregnancy, but a subset of these same women were also enrolled in a cross-sectional study at delivery, the intermittent preventive treatment in pregnancy (IPTp)-MON study.^23^ ^24^ The Asembo Bay ITN cohort (Kenya) identified pregnant women through monthly community census, and the FSP/MISAME (Burkina Faso) study recruited through a community-based network of home visitors.^21^ ^25^ Three studies excluded HIV-infected pregnant women,^22^ ^27^ ^28^ three studies did not assess HIV status^21^ ^30^ ^31^ and the remaining seven studies included both HIV-infected and HIV-negative pregnant women.^10^ ^20^ ^23^ ^25^ ^26^ ^29^ ^32^

The predominant malaria species observed in all studies was *P. falciparum*. The two studies in Papua New Guinea (PNG) also reported *P. vivax* infections.^30^ ^31^ Some studies detected *P. ovale* and *P. malariae* infections but prevalence was very low (<1%). Studies not differentiating between species (n=4) were conducted in areas where *P. falciparum* is the principal malaria species, and *P. vivax* is thought to be largely absent.^20–22^ ^25^ Eleven studies reported high and perennial malaria transmission, while two studies reported medium-to-high perennial malaria transmission.^22^ ^30^

### Measurements

Women in all studies had at least two study visits, at enrolment and delivery, but the frequency and timing of follow-up visits during pregnancy varied by study. Given logistical and computational intensity required in pooling longitudinal measures and substantial heterogeneity in frequency and timing of measurements, only data at enrolment and at delivery were pooled from every study. Tables 2 and 3 show available enrolment and delivery measurements for each included cohort. Measurements included both those variables deemed essential for the main analyses on malaria, anaemia and malnutrition, and those variables deemed optional but informative. A few measurements, specifically malaria infection diagnostics, malnutrition anthropometrics and anaemia diagnostics warrant further discussion.

**Table 2:** Key measures at enrolment across the 13 studies included in the M3 initiative

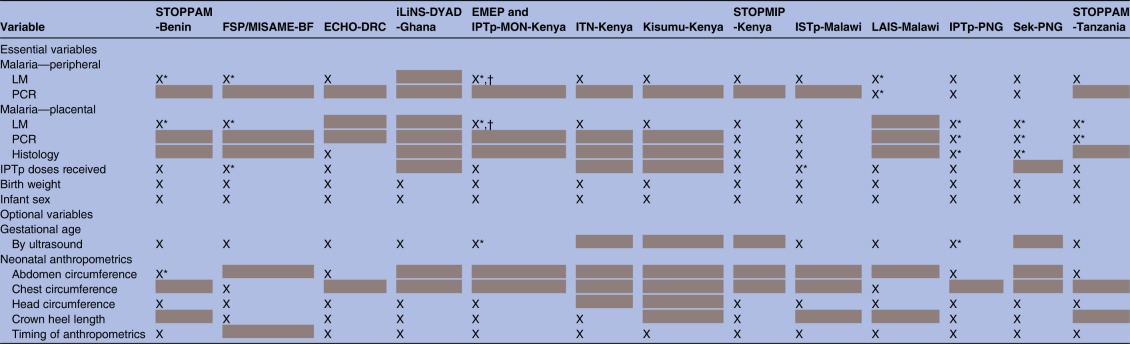

Available information indicated with X, missing data >10% indicated by *, and systematically missing data indicated with grey highlighting.

†The STOPPAM Benin and Tanzania studies primarily used the Parascreen RDT (Zephyr Biomedical Systems, Goa, India), which detects *Plasmodium falciparum* HRP-2 and pLDH.

‡The iLiNS-DYAD-Ghana study used the Clearview Malaria Combo RDT (British Biocell International, Dundee, UK), which detects HRP-2 and plasmodium aldolase.

§Women randomised to the ISTp arms of the STOPMIP-Kenya and ISTp-Malawi studies were tested with a First Response Malaria Ag. (pLDH/HRP2) Combo RDT (Premier Medical Corporation, India).

¶Maternal MUAC measured at delivery.

**Nine studies measured haemoglobin using a HemoCue haemoglobinometer (Hemocue, Angelholm, Sweden),^20–22^ ^26–31^ one study relied on haemoglobin measures from a local laboratory (unknown make),^23^ one study used Sysmex hematological analyser (Kobe, Japan)^32^ and one study collected capillary blood (finger stick) specimens for determination of haematocrit or haemoglobin levels using a standardised chart.^10^ In the ITN-Kenya study, haemoglobin was measured using a HemoCue haemoglobinometer (Hemocue, Angelholm, Sweden) before 1997, and from 1997 onwards capillary blood (finger stick) specimens were collected for determination of haematocrit levels, which were divided by a factor of three and presented as haemoglobin values for consistency with the 1996 data.

BF, Burkina Faso; DRC, Democratic Republic of the Congo; HRP-2, histidine protein-2; IPTp, intermittent preventive treatment in pregnancy; ISTp, intermittent screening for malaria infection in pregnancy; ITN, insecticide-treated bed nets; LM, light microscopy; M3, Maternal Malnutrition and Malaria; MUAC, mid-upper arm circumference; N, no; pLDH, *Plasmodium* lactate dehydrogenase; PNG, Papua New Guinea; RDT, rapid diagnostic test; STOPPAM, Strategies To Prevent Pregnancy Associated Malaria; Y, yes.

**Table 3:** Key measures at delivery across the 13 studies included in the M3 Initiative

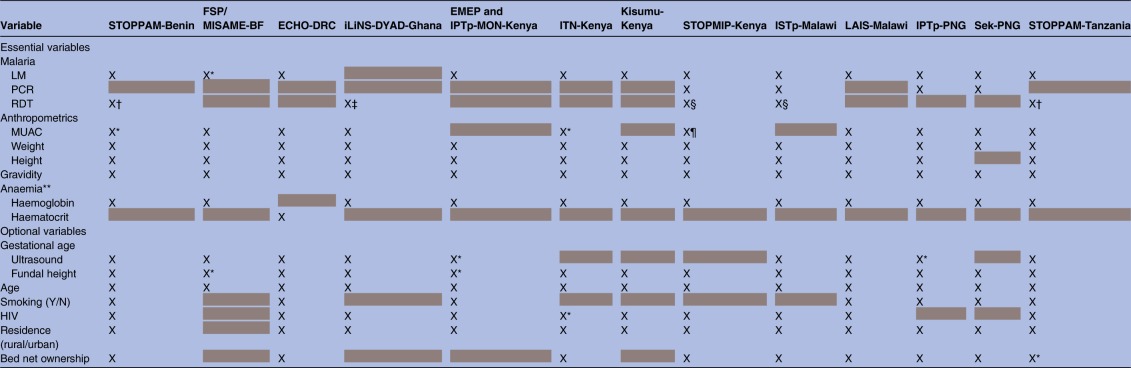

Available information indicated with X, missing data >10% indicated by *, and systematically missing data indicated with grey highlighting.

†For the EMEP/IPTp-MON study, only women co-enrolled in the IPTp-MON study (n=111) had malaria diagnostics at delivery.

BF, Burkina Faso; DRC, Democratic Republic of the Congo; IPTp, intermittent preventive treatment in pregnancy; ISTp, intermittent screening for malaria infection in pregnancy; ITN, insecticide-treated bed nets; LM, light microscopy; M3, Maternal Malnutrition and Malaria; PNG, Papua New Guinea; STOPPAM, Strategies To Prevent Pregnancy Associated Malaria.

#### Malaria infection at enrolment

At enrolment, malaria infection was diagnosed by examination of peripheral blood.^35^ Parasitaemia was assessed using one or more of the three following approaches: LM examination of a Giemsa-stained blood smear, qPCR and RDTs (table 2). In all but two studies, malaria at enrolment was routinely diagnosed by LM. In the FSP/MISAME study in Burkina Faso, LM was only performed if a woman presented with symptoms consistent with malaria, that is, fever or history of fever.^21^ In the iLiNS-DYAD Ghana study, malaria was only diagnosed using an RDT.^22^ RDTs detect circulating target malaria antigens and are increasingly used in malaria-endemic areas when microscopy is not readily available.^35^ ^36^ However, persistence of circulating target antigens after parasite clearance can lead to false-positive test results.^35^ Sensitivity of qPCR is much higher than LM and RDTs, but the role of submicroscopic infections detected by qPCR alone in causing adverse outcomes has not been clearly determined.^37^ ^38^

#### Malaria infection at delivery

In addition to peripheral blood diagnostics at delivery, some studies also assessed placental malaria infection at delivery (table 3). Placental malaria was diagnosed by LM of a placental blood smear, qPCR and/or placental histology.^39^ Three criteria are used to classify placental malaria by histology: presence of infected erythrocytes; haemozoin in monocytes/macrophages and haemozoin in fibrin deposits.^40^ Placental histology is considered the diagnostic reference standard for placental infection.

#### MUAC and BMI

Maternal macronutrient nutritional status was assessed by measurement of women's MUAC and/or BMI at enrolment. MUAC, the circumference of the upper arm, assessed at the mid-point between the elbow and the tip of the shoulder, is frequently used as a broad indicator of maternal protein reserves, fat stores and macronutrient nutritional status.^41^ MUAC was measured in 10 of the 13 studies, which included 61% of our total study population (table 2). Weight and height (to derive BMI) were additionally measured in nine of these studies as well as in the three studies where MUAC was unavailable (table 2). While neither measurement is perfect, MUAC and BMI are easy and low-cost nutritional assessments that can be undertaken in low-resource settings, and are clinically relevant.^42^

#### Anaemia at enrolment

Most women had haemoglobin measurements performed at first ANC, and most received routine iron/folate supplements, although information on their use was not routinely collected. The dosage of folate supplementation (0.4–5 mg) varied by study site.

## Findings to date

Findings from each individual study emphasise how pooling these data has the potential to generate important information on malaria–nutrition interactions in pregnancy.

The ECHO study in the Democratic Republic of the Congo (DRC) was the first study to report an interaction between malaria and malnutrition on the risk of FGR.^10^ In this study, the risk of FGR was 2–8 times higher among women with evidence of malnutrition. In this study, the magnitude of the difference in effect sizes depended on the measurement for malnutrition (BMI vs MUAC vs short stature vs weight gain) and the measurement for malaria (cross-sectional vs cumulative); however, with all measurements of malnutrition and malaria, the pattern and directionality of the modification was consistent. This study, together with a small number of follow-up studies, provided the necessary preliminary data for the M3 initiative.^7^ ^12^

Two participating studies provided evidence regarding nutritional interventions during pregnancy. The FSP/MISAME in Burkina Faso study concluded that multiple micronutrient-fortified food supplementation increased mean birth weight, and that this effect was most pronounced among women who were anaemic or undernourished early in their pregnancy.^34^ The iLiNS-DYAD trial in Ghana found modest overall increases in birth weight with the provision of lipid-based nutrient supplements compared with iron and folic acid supplementation, but showed that the intervention may be most effective when given to primiparous women.^22^ While nutritional assessment was not the primary aim of the remaining studies, the IPTp RCT in PNG and the Strategies To Prevent Pregnancy Associated Malaria (STOPPAM) cohort study in Benin reported associations between low maternal anthropometrics and birth weight.^20^ ^43^

Other studies largely focused on malaria prevention. In the late 90s, the Kisumu cohort study found that implementation of two doses of IPT-sulphadoxine-pyrimethamine (SP) halved the prevalence of placental malaria infection. In another study conducted concurrently in Kenya (Asembo Bay study) use of ITNs was associated with a marked reduction in placental malaria and LBW, but (as expected) with no apparent impact on nutritional status.^25^ The LAIS study, conducted in Malawi from 2003 to 2006, found that adding azithromycin to two courses of monthly SP (SPAZ) was better at reducing malaria at delivery, LBW and preterm birth than monthly SP or two doses of SP.^29^ ^44^ ^45^ SPAZ was also superior when compared with the standard malaria prevention regimen used in PNG (SP plus chloroquine),^30^ where placental infection is an important risk factor for LBW.^31^ The EMEP cohort study found that use of artemisinin combination treatments during the first trimester was not associated with increased risk of miscarriage.^23^ The IPTp-MON study found that with increasing resistance, the efficacy of IPTp-SP in clearing existing infections or preventing new ones is compromised; however, where the sextuple mutant is rare, it remains associated with improvements in birth weight and maternal haemoglobin.^24^

The STOPPAM cohort studies conducted in Benin and Tanzania provided observational data on the effects and potential limitations of two-dose IPTp-SP. Specifically, the STOPPAM-Benin study found that a substantial proportion of women acquired malaria infections late in pregnancy, sometimes after the second IPTp dose had been given.^20^ The STOPPAM-Tanzania study also found that malaria infections in the first or second trimester were associated with altered fetal growth that might not be detectable until the third trimester.^32^ Trial data from the FSP/MISAME study further corroborated these observational studies; the FSP/MISAME study found that malaria infection during the first trimester was associated with higher risk of LBW and that additional doses of IPTp might be more effective.^21^ ^46^ These studies, along with others, have highlighted the importance of early and frequent malaria prevention to improve birth outcomes, and IPT-SP is now routinely given monthly until delivery.^47^ ^48^

The intermittent screening for malaria infection (ISTp) Malawi study assessed another malaria prevention strategy by comparing IPTp-SP with ISTp at ANC visits using RDTs and treating infected women with dihydroartemisinin-piperaquine (DHA-PQ).^28^ The STOPMIP Kenya study also assessed ISTp with DHA-PQ, comparing it to IPTp with DHA-PQ as well as IPTp-SP. Analyses from both studies suggest that ISTp-DHA-PQ is not superior to the existing strategy of IPTp-SP, although IPTp-DHA-PQ was more effective than both ISTp-DP and IPTp-SP.^27^

## Future plans

We plan to assess whether maternal macronutrient nutritional status, as assessed by MUAC, BMI and maternal height, alters the risk of reduced birth weight that is associated with malaria infection during pregnancy. Among women who underwent a dating ultrasound scan in early pregnancy, analyses will be performed using preterm birth and small-for-gestational age as end points in addition to LBW. To inform policy decisions, we further plan to use advanced epidemiological methods to stochastically model the impact that plausible targeted antimalarial and nutritional interventions during pregnancy could have on the number of babies born LBW in malaria endemic areas.^49–51^ Additional future analyses include, but are not limited to, the following: the impact of anaemia on LBW; the interaction between anaemia and malaria with regard to increasing the risk of LBW; the mediation of the effect of malaria through anaemia on LBW; and the usefulness and agreement of MUAC versus BMI to predict adverse birth outcomes. Short maternal stature, potentially indicative of stunting and reduced adolescent catch-up growth (chronic undernutrition), has also been associated with increased risks of adverse birth outcomes and will be explored using this pooled data set.^52^ Finally, we will assess aforementioned relationships and effects among pregnant women with HIV, a patient cohort known to be at increased risk of adverse outcomes due to malaria and malnutrition.^53^

## Strengths and limitations

This pooled birth cohort holds promise as the largest pregnancy data set to date to address questions regarding malaria and nutrition that are of great importance to maternal and infant health. Prior research on this topic has been limited by insufficient statistical power to examine the interaction between malaria and malnutrition. The pooled M3 offers the opportunity to overcome this limitation by increasing statistical power and precision, which is particularly valuable for conducting subgroup analyses and investigating interactions. The substantial size of the pooled data set also facilitates the application of rigorous methodological approaches for systematically missing data, such as multiple imputation by chained equations, standardisation, latent variable methods or transformation of measurements to create commensurate measures.^54^ ^55^ Availability of individual-level data, rather than aggregate data from each study, allows definitions of exposures and outcomes to be harmonised. Individual-level information on malaria and malnutrition further enables implementation of complex modelling to assess targeted antimalarial and nutritional interventions during pregnancy. The diversity of study populations included in the M3 cohort, for example, with respect to location, ethnicity, malaria transmission intensity, malaria and nutrition interventions, health infrastructure and HIV prevalence, could provide important data with regard to generalisability of our findings. Specifically, this level of population diversity, coupled with availability of data on multiple risk factors for LBW and greater statistical power to assess subgroup effects, may allow the identification of high-risk women who could benefit most from more intensive antenatal care, nutritional supplements and alternative malaria prevention regimens, and of the settings in which these interventions would be most effective.

The main limitations of the pooled data set are related to missing data and heterogeneity of the collected data. First, information that was collected by all studies varied, leading to systematically missing data on a number of important variables (tables 2 and 3). The limited availability of accurately measured gestational age hinders the ability to conduct preterm birth and small-for-gestational age analyses for the entire data set (table 3), but should allow for meaningful subanalyses. Second, exclusion of miscarriages and stillbirths may cause selection bias (left truncation) for some analyses, given the possibility of differential rates of early pregnancy losses in relation to infection and nutritional status.^56^ We will assess this potential bias using data on miscarriages and stillbirths extracted from a subset of studies.^21^ ^23^ ^27^ ^28^ Third, potential confounders such as concomitant helminth infection and micronutrient deficiencies were not measured in participant studies.^57^ ^58^ Fourth, while MUAC only modestly changes throughout pregnancy, BMI tends to increase with gestational age: gestational age-adjusted BMI may be one approach to address this problem. Fifth, while we have information on HIV status for a majority of studies, information on use of antiretroviral therapy (ART) was not extracted, and secular changes over time regarding use and efficacy of ARTs could influence the comparability of results across studies that enrolled HIV-infected women. Finally, since the intention was to concentrate on areas of moderate-to-high *P. falciparum* transmission, we did not include any studies representing other malaria-affected regions such as Central/South America and India.

## Collaboration

The investigators welcome collaboration for future projects and invite proposals for additional analyses. Further details can be obtained by contacting SR (sroger@unimelb.edu.au) and HWU (hwunger@doctors.org.uk).
